# Sharing of hand kinematic synergies across subjects in daily living activities

**DOI:** 10.1038/s41598-020-63092-7

**Published:** 2020-04-09

**Authors:** Verónica Gracia-Ibáñez, Joaquín L. Sancho-Bru, Margarita Vergara, Néstor J. Jarque-Bou, Alba Roda-Sales

**Affiliations:** Department of Mechanical Engineering and Construction, Universitat Jaume I, Castelló, Spain

**Keywords:** Computational biology and bioinformatics, Computational biology and bioinformatics, Biomedical engineering, Biomedical engineering

## Abstract

The motor system is hypothesised to use kinematic synergies to simplify hand control. Recent studies suggest that there is a large set of synergies, sparse in degrees of freedom, shared across subjects, so that each subject performs each action with a sparse combination of synergies. Identifying how synergies are shared across subjects can help in prostheses design, in clinical decision-making or in rehabilitation. Subject-specific synergies of healthy subjects performing a wide number of representative daily living activities were obtained through principal component analysis. To make synergies comparable between subjects and tasks, the hand kinematics data were scaled using normative range of motion data. To obtain synergies sparse in degrees of freedom a rotation method that maximizes the sum of the variances of the squared loadings was applied. Resulting synergies were clustered and each cluster was characterized by a core synergy and different indexes (prevalence, relevance for function and within-cluster synergy similarity), substantiating the sparsity of synergies. The first two core synergies represent finger flexion and were present in all subjects. The remaining core synergies represent coordination of the thumb joints, thumb-index joints, palmar arching or fingers adduction, and were employed by subjects in different combinations, thus revealing different subject-specific strategies.

## Introduction

Human hand kinematics is extremely complex due to the high number of degrees of freedom (DoF) involved. However, these DoF are commonly used interdependently, both between joints within a finger^1–3^ and between joints of different fingers^2–4^, the correlation being higher for closer joints. This interdependency has been suggested as a strategy of the motor system, which may use kinematic synergies to simplify the control of the hand^5,6^. Different works have studied the postural synergies during static hand postures in grasp^7,8^, performance of letters of the sign language alphabet^9^, reach-to-grasp movements^2,10^, object manipulation^11,12^ or in real daily activities^3^. Some of these studies came to the conclusion that the kinematic synergies are both task-dependent and subject-dependent^12^: a given subject is expected to use different grasps to perform two different tasks; and for a given task, two different subjects may engage different strategies. Conversely, synergies found in many studies, with different tasks and subjects, were quite similar^3,8,11,13–15^, which seems to contradict the previous statement. A recent work^16^ suggests that synergies used by the subjects are intrinsically sparse both in DoF and in actions (i.e., each synergy uses a limited set of DoF and each action is implemented with a combination of a limited number of synergies) and that there is a large set of synergies, shared across subjects. Confirming such hypothesis, and identifying these sparse synergies underlying the hand function would be of great help in several research fields: rehabilitation strategies, clinical decision-making or in prostheses design. However, the characterization of the sparse synergies shared across subjects in their daily activities remain unveiled because of poor representativeness of the existing studies in the number of subjects considered and the tasks analysed (static grasp postures or few simple tasks), but also because of the methods used.

Statistic methods for dimensional reduction are used to search synergies, Principal Component Analysis (PCA) being the most widely applied since it has provided good results in hand kinematics^17^. PCA allows the original multivariate space of highly correlated variables (DoF) to be transformed into a smaller set of new uncorrelated variables (linear combinations of the original variables called synergies) explaining a high percentage of the original variability. The obtained synergies with PCA depend on the selected tasks^12^ and the subjects participating in the experiment, but also on the way PCA is applied (as discussed later), so that interpretation and comparison of synergies found in literature should be made with caution.

First, PCA can be applied globally (to the recorded kinematics of different subjects altogether) or per subject (one PCA to the kinematics of each subject). However, we hypothesise that if PCA is applied to data from several subjects together, merged unreal synergies might appear, as different subjects may engage different strategies to perform a given task. In most previous works, PCA has been applied globally to the kinematic data of different subjects^8,13^. If our hypothesis is true, inferring single-person coordination patterns from those results may lead to unrealistic results, and in order to analyse real subject-specific coordination patterns, PCA should be applied per subject^11^.

Second, the synergies in PCA can be the eigenvectors of either the correlation matrix (CoM) or the covariance matrix (CvM), ordered according to the magnitude of the variance explained. PCA using the CvM, is quite sensitive to the variances of the original variables so that those with larger ranges of variation dominate over those with small ranges. In order to make that each variable contributes equally to the analysis, PCA may be applied to the CvM with standardized data (mean = 0 and SD = 1 for each DoF)^2,18^, which is equivalent to applying PCA to the CoM^3,12^. This standardization is commonly applied in in PCA, but is rarely detailed in methods^8,12^. Furthermore, this standardization is not physiologically coherent, as it could imply scaling with different maximal values (ranges of motion) for a same subject depending on the tasks performed. Analogously, the range of motion used when performing a set of tasks may differ also significantly among subjects, and consequently standardization will also differ across subjects (subjects may engage different strategies when performing a given task). In other words, synergies obtained with this standardization are dependent on differences in the joint ranges of motion of the recordings, making synergies non-comparable. Furthermore, this standardization procedure may introduce errors if a joint is kept approximately unchanged during the recordings, thus experiencing a fairly null range of motion (e.g., tasks performed using just the thumb and index finger, or a patient with his thumb carpometacarpal joint practically constrained), which would result in noise magnification. In conclusion, enabling the comparison of synergies from different tasks and subjects requires considering, for a given joint, the same not null scaling for all subjects and tasks, independently of the sample data recorded, and with physiological meaning. However, such scaling will result into non-standard data (i.e. mean=0, SD = 1 is not accomplished).

This work proposes the use of maximum active ranges of motion as fixed values to scale each DoF, regardless of the recorded sample of postures to which PCA is applied, so that coordination of the DoF with the lowest ranges of motion is not hidden, but comparison between subjects and tasks is still feasible, and with no noise magnification.

Finally, most works in the literature do not look for synergies sparse in DoF. Actually, some studies^16^ suggest that the sparsity in DoF of synergies cannot be addressed by PCA methods because all DoF are used to compute any synergy. Although this is true for standard PCA, sparsity in the DoF of synergies can be addressed by applying a rotation method trying to minimize the number of DoF in each synergy. In this sense, Varimax rotation maximizes the sum of the variances of the squared loadings, so that any given synergy comprises just a few DoF with very high loadings on this synergy, while the remaining DoF have near-zero loadings. Despite the existence of other methods for obtaining sparse synergies^16,19^, PCA with Varimax rotation has the advantage of the simplicity of the method and the low time of computation. However, it has been scarcely used in literature^13^.

Therefore, considering all the aforementioned, and with the aim of substantiating the existence of sparse grasping synergies, we propose the look for the set of sparse synergies shared across subjects by: (i) applying PCA per subject, with the new scaling method proposed and applying Varimax rotation, to the kinematics recordings of a previous work^20^, where a sample of healthy subjects performed a wide set of real activities of daily living (ADL) representative of healthy hand function^3^; and (ii) checking for similitudes across comparable subject-specific synergies obtained, in order to detect whether subjects perform ADL by using similar synergies, what those synergies are, and their frequency of use by the subjects. Additionally, we have investigated whether merged unreal synergies appear if PCA is applied to data from several subjects altogether.

## Methods

### Experiment

Data used in this analysis are those from a previous work from the authors^20^, where the hand kinematics of twenty-four right-handed adults were obtained while performing a representative set of ADL (Table 1) for the purpose of computing functional ranges of motion. The experiment performed to obtain these recordings is briefly explained next. All the subjects gave their informed consent to participate in the experiment (approved by the University Ethics Committee – *Comissió Deontològica*), and specific informed consent for publication in an online open-access publication have been obtained for photos that could lead to identification of the participant. The subjects (12 females, 12 males) were free from hand pathologies or injuries, and were selected to be representative of adult population hand sizes: mean (SD) hand length and hand width, 186.0 (11.3) mm and 81.6 (6.5) mm, respectively. Ages were limited to 50 years to avoid kinematic alterations due to aging.

**Table 1:** ADL recorded in the experiment, and corresponding ICF chapter.

ICF chapter	ADL selected
3. Communication	1. Reading
	2. Writing
	3. Speaking by phone
	4. Dialling numbers on the phone
	5. Writing using the keypad
4. Mobility	6. Handling a book
	7. Unlocking a door with a key
	8. Opening a door
5. Self-care	9. Turning a faucet on and off
	10. Washing and drying hands
	11. Cleaning teeth
	12. Putting toothpaste on a toothbrush
	13. Combing hair
	14. Putting a shirt on and doing buttons up
	15. Putting pants on, doing up button and zipper
	16. Putting shoes on and tying laces
	17. Eating soup
	18. Cutting with a knife
	19. Eating with a fork
	20. Pouring water
	21. Drinking water
6. Domestic life	22. Using a spray
	23. Cleaning with a cloth
	24. Ironing

**Figure 1:**
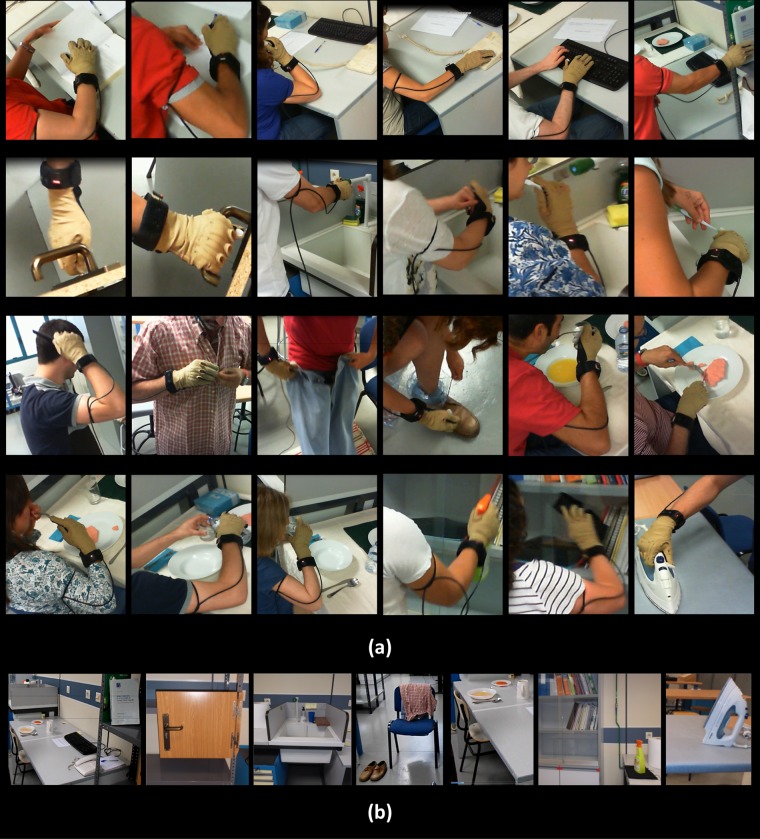
(**a**) Stills from the task execution, one for each ADL performed (Table II) and (**b**) images of the experimental scenarios with main real objects used.

WHO’s International Classification of Functioning, Disability and Health (ICF), the only reference recognized worldwide to assess health aspects^21,22^, was used to select a representative set of ADL within the ICF chapters requiring the use of the hand^23^ (Table 1 and Fig. 1). Each subject performed these 24 ADL in laboratory conditions following precise instructions on how to accomplish each ADL, and using real objects. As an example, for the action of drinking water, the subject sat in front of a table, with his/her hands lying on the table shoulders’ width apart, and the position of the glass half full of water was the same for all the subjects. When indicated to do so by the operator, the subject took the glass, drank water for three seconds, put the glass back in its original position, and returned his/her hands to the starting position lying on the table. The kinematics of the right hand were recorded (75 Hz) during the performance of these ADL using an instrumented glove (Cyberglove Systems LLC; San Jose, CA) to obtain 16 joint angles^24^: metacarpophalangeal flexion (MCP1 to MCP5, 1 to 5 meaning thumb to little digits), interphalangeal flexion of the thumb (IP1), proximal interphalangeal flexion of the fingers (PIP2 to PIP5), flexion and abduction of the carpometacarpal joint of the thumb (CMC1), relative abduction between finger MCPs (index-middle, middle-ring and ring-little), and palmar arching. The recordings were filtered with a 2nd-order 2-way low-pass Butterworth filter with cut-off frequency of 5 Hz.

### Data analysis

First, the appropriateness of the PCA was checked by means of Bartlett’s test of sphericity^25^. Second, original data were scaled with a non-standard scaling based on active range of motion data in order to have the same data scaling in all subjects. In this way, the subjects’ PCs (synergies for each subject in form of vectors) would be comparable among them and to those of other subjects, regardless of the ranges of motion of their particular recordings. The way of applying this scaling is by subtracting the mean and dividing by the SD as it is commonly done in normal standardization, but instead of using mean and SD from the sample, mean and SD used are those from Table 2. In a previous work^20^, the authors recorded static postures to obtain the maximal active ranges of motion of each joint in a sample of healthy subjects, and these extreme values were averaged across subjects. Mean and SD taken from these extreme postures are reflected in Table 2.

**Table 2:** Mean and SD values per joint to apply the non-standard scaling.

Digit	1				2–3	3–4	4–5	Palmar Arch
Joints	CMC A	CMC F	MCP F	IP F	MCP A			
Mean	7.8	8.0	2.6	18.8	7.3	4.9	5.4	41.1
SD	16.9	48.3	33.3	79.4	39.5	29.5	32.5	69.1
Digit	2	3	4	5	2	3	4	5
Joints	MCP F	PIP F						
Mean	22.7	27.0	25.3	23.3	52.5	45.0	46.5	41.1
SD	67.8	77.6	68.4	63.9	79.6	73.0	79.7	69.1

Digits 1 to 5 mean thumb to little digit. F for flexion, A for abduction.

Then, PCA^26,27^ was applied to the 16 joint angles measured in all the recordings (24 recordings, one per ADL) of each subject after applying the non-standard scaling, in order to identify the subject-specific kinematic synergies. The covariance matrix was computed, and the eigenvalues (variance explained) and eigenvectors (principal components) computed. The first four synergies were computed for each subject. To ensure that all the ADL had the same weight, the number of frames of each recording was previously resampled to 1000 frames. Varimax rotation was finally applied. This rotation^13^ maximizes the sum of the variances of the squared loadings, so that any given PC comprises just a few variables with very high loadings on this PC, while the remaining variables have near-zero loadings, so that sparsity in the DoF of synergies is considered even using PCA.

The resulting subject-specific synergies of the 24 healthy subjects were clustered according to their similarity. This was addressed through a hierarchical cluster analysis (grouping metric: absolute value of cosine; method: complete linkage, i.e., farthest neighbour distance), so that it considers similarity of synergies through the angle between synergies. Note that the absolute cosine value has been used to ensure the same proximity for opposite synergies (vectors), as their orientation is arbitrarily assigned in the mathematical procedure of PCA. The cluster selection criterion was the minimum number of clusters ensuring that no cluster contains more than one synergy from the same subject. A description of each cluster is presented through the representative coordination of the cluster, percentage of subjects with a synergy in the cluster, variance explained by the synergies of the cluster, level of similarity of the synergies within the cluster, and simultaneous appearance of the synergies per cluster in the same subjects.

The coordination of each cluster was interpreted from the loadings of a representative synergy, called core synergy (CS) hereafter. These CSs may be considered as the core coordination patterns used by the healthy subjects in ADL. To obtain these CSs, first the synergies within a cluster were reversed (the sign of the loadings was changed) whenever necessary so that they all had the same orientation (positive cosines among all the synergies in the group). Then, synergies of each cluster were averaged to obtain the CSs. To graphically represent the CSs with Opensim, we constructed hand postures by adding the CS scaled by a constant factor to the mean conformation, the factor acting to constrain the range of motion within biologically feasible limits according to^20^.

The level of similarity of the synergies within each cluster was analysed both in angles and in loadings. In angles, they were analysed through the mean and SD values of the angles between the synergies of the cluster and the corresponding CS. In loadings, the analysis was performed through the mean and SD values of the root mean square deviation (RMSD) between the loadings of the synergies within a cluster and those of the corresponding CS.

Finally, PCA was applied in the same way but globally to all subjects to check the hypothesis that if PCA is applied to data from several subjects together, merged unreal synergies might appear.

### Ethical approval and consent to participate

All procedures performed in studies involving human participants were in accordance with the ethical standards approved by the ethics committee of our University and with the 1964 Helsinki declaration and its later amendments or comparable ethical standards.

## Results

Bartlett’s test of sphericity confirmed the suitability of the 24 samples on which PCA was applied (one per subject) for the application of this statistical method (p < 0.01 in all cases). The 4 PCs obtained for each healthy subject (subject-specific synergies) accounted for a mean variance of 77.3% (SD 1.9%). All of them corresponded to eigenvalue>1. The resulting 96 PCs were sparse in DoF, with only 25% of loadings greater than 0.25.

**Figure 2:**
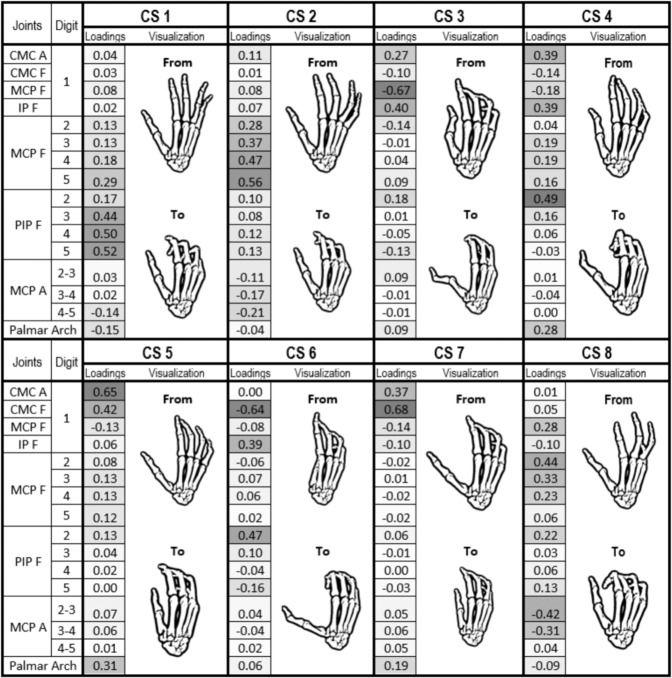
CS loadings along with their Opensim representation ordered by percentage of healthy subjects in each cluster and mean percentage of variance explained per cluster. CSs were graphically represented using the hand kinematic model developed in Opensim by the ARMS lab of the Rehabilitation Institute of Chicago^28^. Digit 1 to 5: thumb, index finger, middle finger, ring finger and little finger. Joints: CMC (Carpometacarpal), MCP (Metacarpophalangeal), IP (Interphalangeal), PIP (Proximal Interphalangeal). Joint suffix: A (Abduction), F (Flexion).

**Figure 3:**
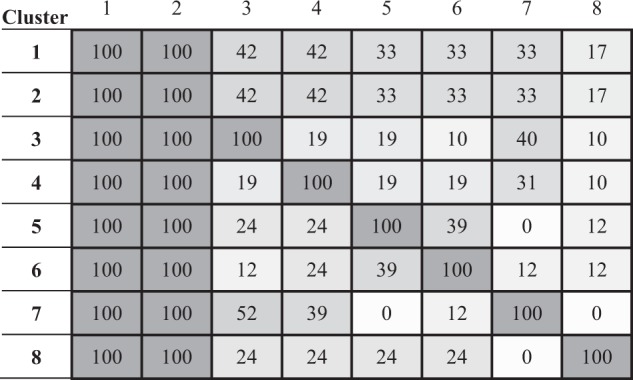
Index of simultaneity. Percentages of subjects who, while having a subject-specific synergy in a cluster i (row i), have a subject-specific synergy in another cluster j (column j).

**Figure 4:**
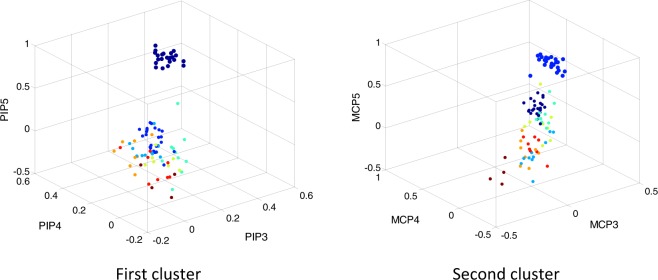
3D visual representation of the first two clusters as an example. The 3 main loadings representing the cluster are represented for each CS. Loadings of each group are represented in a different colour.

The hierarchical cluster analysis yielded eight clusters. Figure 2 shows the CSs representing each cluster (with only 19% of the values greater than 0.25). The percentage of healthy subjects presenting that specific coordination, variance explained by the synergies of the cluster, and level of similarity of the synergies within the cluster are shown in Table 3. The simultaneity of appearance of synergies in healthy subjects is presented in Fig. 3. To make results clearer, as a matter of example, in Fig. 4, a visual representation of the first two clusters, plotted in a 3D space, is represented by showing the three main loadings defining the cluster coordination.

**Table 3:** Statistics values: % subjects (percentage of subjects presenting the coordination), % variance (mean and standard deviation (SD) variance of the synergies within the cluster, Level of similarity in angles (mean and SD of angles formed between each PC of the cluster and the CS of the corresponding cluster) and in loadings (mean and SD values of the root mean square deviation (RMSD) between the loadings of the synergies within a cluster and those of the corresponding CS).

Cluster characteristics	CS 1	CS 2	CS 3	CS 4	CS 5	CS 6	CS 7	CS 8
% subjects	100%	100%	42%	42%	33%	33%	33%	17%
% variance: Mean (SD)	28% (5%)	26% (5%)	13% (2%)	13% (3%)	13% (3%)	11% (3%)	10% (1%)	11% (1%)
Level of similarity in angles: Mean (SD)	13° (3°)	17° (6°)	26° (8°)	25° (9°)	26° (6°)	22° (7°)	34° (8°)	24° (8°)
Level of similarity in loadings: Mean (SD) of RMSD	0.06 (0.01)	0.07 (0.03)	0.11 (0.03)	0.10 (0.04)	0.11 (0.02)	0.09 (0.03)	0.14 (0.03)	0.10 (0.03)

CS1 and CS2 were present in all the healthy subjects, together explaining 54% of variance, and with a high level of intra-cluster similarity. These two core synergies only involve movement of the fingers. CS1 shows a coordinated flexion of the proximal interphalangeal (PIP) joints of fingers, increasing from middle to little fingers, with flexion of the MCP joint of the little finger. CS2 is a coordinated flexion of the MCP joints of fingers, increasing from index to little fingers.

CS3 to CS7 represent different coordinations of the carpometacarpal (CMC) joint of the thumb with other joints (from the thumb itself, from the index finger or with palmar arching). CS3 mainly involves thumb joints and represents CMC abduction with MCP extension and interphalangeal (IP) flexion, present in 42% of the population, with a medium level of intra-cluster similarity. CS4 represents a coordination between the thumb (CMC abduction and IP flexion) and the index finger (PIP flexion), along with a slight palmar arching. CS6 also represents coordination of index and thumb, but in this case flexion of thumb IP and index PIP are coordinated with thumb CMC extension. Both CS5 and CS7 core synergies (mutually exclusive) represent opposition of the thumb, but with slight differences: in both synergies the thumb is opposed through CMC flexion and abduction, but CS5 with more CMC abduction, while CS7 with more CMC flexion. CS5 coordination is also accompanied by palmar arching (somewhat present in CS7).

CS8, the least frequent coordination in healthy subjects (17% of the population), represents mainly a coordinated flexion of the MCP joints of the index and middle fingers, along with a high adduction of the index-middle and middle-ring fingers. MCP flexion of the thumb is also present.

As regards simultaneity, two out of the four subject-specific synergies correspond to CS1 and CS2. When coordination of index and thumb is present in a third subject-specific synergy, through CS4 or CS6, the fourth subject-specific synergy is mainly a coordination of flexion-abduction of thumb CMC, through CS7 and CS5 respectively. That is, when CS4 is present, the fourth coordination is mainly CS7, and when CS6 is present, the fourth coordination is mainly CS5. However, when CS3 is present (CMC abduction coordinated with thumb MCP extension and IP flexion), the fourth coordination is predominately CS7, i.e., thumb CMC flexion with some abduction.

**Figure 5:**
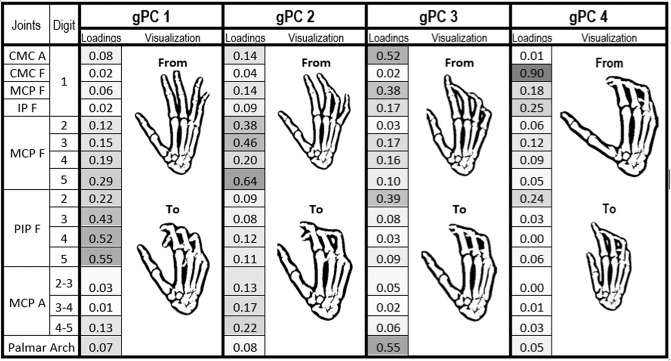
Global PCs (gPC) loadings along with their Opensim representation ordered by variance explained (24% for gPC 1, 23% for gPC 2, 13% for gPC 3, 10% for gPC 4). gPCs were graphically represented using the hand kinematic model developed in Opensim by the ARMS lab of the Rehabilitation Institute of Chicago^28^. Digit 1 to 5: thumb, index finger, middle finger, ring finger and little finger. Joints: CMC (Carpometacarpal), MCP (Metacarpophalangeal), IP (Interphalangeal), PIP (Proximal Interphalangeal). Joint suffix: A (Abduction), F (Flexion).

The synergies obtained when applying PCA globally can be observed in Fig. 5. The first two synergies only involve movement of the fingers. The first global PC (gPC 1) shows a coordinated flexion of the proximal interphalangeal (PIP) joints of fingers, increasing from middle to little fingers, with flexion of the MCP joint of the little finger. gPC 2 is a coordinated flexion of the MCP joints of fingers, increasing from index to little fingers, except for the ring finger. gPC 3 represents coordination of palmar arching, thumb abduction, thumb MCP flexion and index PIP flexion, while gPC4 represents thumb CMC flexion with a slight thumb IP flexion.

## Discussion

The underlying subject-specific synergies during the performance of a set of representative functional ADL in a sample of 24 healthy subjects have been obtained. A total of 96 subject-specific synergies were identified (4 synergies per subject), accounting for a mean total variance of 77.3%, therefore representing the gross motion of the hand in ADL performance. The PCs that were discarded may be potentially representative of subtler movements, but they could also correspond to noise.

The idea that there is a large set of synergies, shared across subjects, and that for a same task each subject maps out different strategies from among several feasible ones^16^ has been investigated through a hierarchical cluster analysis. The 96 subject-specific synergies were clustered into eight groups representing similar coordinations. Interestingly, two of the groups include synergies from all subjects. Therefore, this study partially confirms this idea of sparsity, given that all subjects invariably seem to use two of the synergies. The rest of the synergies seem to depend on the different strategies employed by each specific subject.

In addition, synergies found are in accordance with the idea of sparsity introduced in recent works^16^, since they involve a limited set of DoF thanks to the Varimax rotation. Prevete *et al*.^16^ argued that PCA is not a reliable method to look for sparse synergies because it considers all DoF in the computation. However, as can be seen in the results from this study, using PCA with Varimax rotation means that each synergy obtained comprises just a limited number of DoF with very high loadings on this synergy, while the others remain with almost zero loadings. In previous studies, Varimax rotation was not commonly used, and consequently all DoF were merged in a same synergy, as in Santello *et al*.^8^, where first and second PCs include loadings of all DoF from all fingers, or in Thakur *et al*.^11^, where the same effect can be seen especially in the first PC. In those studies that used Varimax rotation for the purpose of clarifying interpretation of the results^13,14^, sparsity in the DoF was observed, despite not being the goal pursued.

Coordination represented by each cluster has been interpreted from the loadings of the corresponding CS, obtained by averaging the subject-specific synergies within the cluster. The resulting CSs, despite not being strictly attributable to any subject, do not represent fake strategies, given that the averaged loadings within the group are very similar. The first two CSs, representing coordinations of flexion motion at finger joints, clearly explain more variance than the others and are shared by all the subjects when performing ADL. Therefore, these first two CSs are very consistent among subjects, in line with previous works^3,8,11,13,14^, in spite of the different methods applied. The remaining CSs correspond to coordination of thumb, thumb and index or palmar arching, and are more variably used by subjects in the performance of ADL. These results would explain the control paradigm of global muscle coordination as a combination of lower order control of individuated muscles and higher order control of robust subject-specific muscle synergies^29^, although recent works point to the fact that EMG activity may not necessarily reflect neural drive^30^. Furthermore, these results highlight the importance of the independence of the thumb and index finger to perform ADL, in accordance with the literature^3,18,31^. High-order synergies - i.e. synergies associated to the 3^rd^ and next PCs and explaining a smaller percentage of variance^8^ - found in previous works also refer to the coordination of the thumb, or thumb and index, which would be consistent with thumb independence, but in many cases the thumb also appeared coordinated in the first synergies with the rest of the digits^8,13,15^. Higher-order synergies referring to the thumb might be representing fake strategies when PCA is applied globally, as in Jarque-Bou *et al*.^13^, where thumb CMC abduction appears independently to the rest of the thumb joints or even to the index joints, unlike what happens in this work.

On applying PCA per subject instead of globally, the real strategies performed by each subject, not reported in previous works, are unveiled. From the comparison of the synergies computed globally (Fig. 5) with the core synergies obtained (Fig. 2) it can be observed how the first two synergies are highly similar, corresponding to flexion of the fingers. However, the high-order global synergies reflect a kind of combinations of some of the high-order subject-specific ones or new coordinations, not revealing the strategies followed by subjects. Thumb abduction is coordinated with thumb MCP flexion and index PIP flexion, along with palmar arching for the third gPC, being as a king of combination of the core strategies CS3, CS4 and CS5. Thumb CMC flexion is coordinated with a slight index PIP flexion in the fourth gPC not corresponding to any global synergy. Hence, if real strategies followed by subjects are aimed, PCA must be applied per subject.

However, even when subject-specific synergies are found, they might be hiding some strategies if no scaling is used or even if common standardization is applied (mean = 0, SD = 1), as in Ingram *et al*.^3^, where abductions are imperceptible. Scaling original data helps avoiding the DoFs with the lowest ranges of motion to be hidden, but with common standardization, synergies obtained depend on the range of motion of the recordings and noise can be magnified. Thanks to the new scaling used, based on the active range of motion per joint of a generic sample of subjects, the subject-specific synergies are comparable between subjects, even for subjects not included in the sample where PCA has originally been applied and no errors of noise magnification are avoided. The new scaling proposed to obtain comparable subject-specific synergies unveils the different healthy strategies in fine manipulation, reflected through the different coordinations of the thumb and thumb-index used by each subject. It can be observed that coordination of thumb and index (CS4 or CS6) appears along with thumb opposition (CS7 or CS5). The difference between the two synergies of index-thumb coordination (CS4 vs. CS6) is related to using more thumb CMC abduction (CS4) or thumb CMC flexion (CS6). Likewise, the difference between the two synergies of thumb opposition (CS7 vs. CS5) is related to using more thumb CMC abduction (CS5) or thumb CMC flexion (CS7). It seems that when a subject use more thumb CMC abduction for opposition, he/she tends to use more thumb CMC flexion for thumb-index coordination.

Regardless of the differences in the method applied (rotation, global/individual synergies or scaling), the synergies found in many previous studies present a similar structure, with the first two synergies representing gross accommodation of digits and higher-order ones representing fine manipulation. Notwithstanding, differences in the synergies can be observed. Besides differences due to the method for extracting the synergies, there are other sources of differences. Obviously, the more DoF considered, the higher the number of synergies found, as in those that include distal interphalangeal (DIP) joints of fingers^11,13^ or the wrist^14^. The additional joints considered may move coordinately with the other DoF, giving rise to differences not only in the number of synergies but also in their configuration. In Thakur *et al*.^11^, who considered the unconstrained haptic exploration task of several everyday objects and measured flexion of finger DIP joints, unlike this work, these joints appeared coordinated to the PIP and MCP ones, as in other works on static grasping postures^13^. In Thakur *et al*.^11^, thumb coordination appeared more appreciatively from the third coordination onwards and, additionally, their last coordination refers to finger adduction, as in our work. This coordination appeared in a higher order synergy than in our study – the ninth. In a recent work^14^ that analysed grasping tasks in a high number of subjects and covering a wide range of joints (including the wrist), the number of synergies found was higher. However, disregarding those that include the wrist, the structure of the synergies was similar to that obtained in this study.

In addition, dissimilitude in the synergies observed with respect to those obtained in other studies may arise because of the actions considered in each study, as synergies are task-dependent^12^. This is probably the cause of the observed coordination in the thumb between MCP extension and IP flexion in our study. Such coordination appears during real ADL when performing thumb-index pinch and some force is required, but it does not appear in previous works with imaginary grasping postures or in haptic manipulation, in which no force is applied. As a consequence of this task-dependence, synergies are highly affected by the level of reality of the actions performed. This may explain why Santello *et al*.^8^, analysing static imaginary grasping postures, extracted only two synergies, which explained far more variance than ours (84% vs. 54%), as the postures analysed corresponded to free movement, without object interaction. The number of synergies obtained by Thakur *et al*.^11^ was higher than that obtained by Santello *et al*.^8^ for the same variance explained, since they considered manipulation, requiring a greater variety of postures. The performance of real ADL includes reach-to-grasp, release, and manipulative grasping movements, which provide a higher variability of postures than when performing just one static posture representative of the whole action, thus leading to more synergies.

To sum up, the highest similarity to our results corresponds to those studies where the DoF analysed are similar to ours, to those where wider ranges are covered because of the postures/movements analysed, so that the impact of the normalization is lower, and to those studying real manipulation of objects, either in haptic exploration^11^ or in real ADL performance^3^.

Although common statistic techniques (Principal component analysis and clustering) have been used to look for the set of synergies shared across subjects, the novelty introduced in the scaling proposed overcomes problems due to noise magnification, and allows comparability in subject-specific synergies, which are much more realistic than global synergies. The use of Varimax rotation overcomes the problem of sparsity in DoF. Other methods could be used to overcome the problem of sparsity^16^: non-orthogonal rotation^19^, avoiding the orthogonality restriction that could be introducing a slight unrealism, or non-negative matrix factorization^32^. However, non-negative matrix factorization would have required weird reference postures to ensure all joint angles as positive, and non-orthogonal rotation does not preserve the independence of the factors and the interpretation of the factorial space becomes less obvious.

In addition, despite the fact that synergies are task-dependent, the level of reality and the extension of this study (twenty-four healthy subjects performing twenty-four carefully selected ADL) makes it the most representative of functionality to date, providing a novel insight into healthy kinematic characterization in ADL through the underlying functional synergies and their frequency of appearance. The frequency of each kinematic synergy, and the relationship with the muscle synergies obtained in previous works^29,33,34^ could be used to achieve a more intuitive control of hand prostheses, but also in hand kinematic dysfunction evaluation, going beyond the current analysis of range of motion^35^. A recent study proposed to define healthy motion patterns^36^ for a future clinical application. According to the results of this study, one important indicator of dysfunction may be the absence of the main finger synergies (CS1 or CS2), as this would imply the patient’s inability to perform a coordination present in all healthy subjects. Another indicator may be the presence of synergies different from those of healthy subjects, as this would show the patients’ need to replace coordinations they are unable to perform.

## Conclusion

The space of the core subject-specific kinematic hand synergies (CSs) underlying the performance of a wide set of representative ADL has been provided. The percentage of subjects presenting each of these CSs represents the frequency of appearance of each synergy. This makes it highly applicable in different fields that attempt to understand the behaviour of the human hand in ADL, such as the design of products for everyday life, the design and control of hand prostheses, in clinical decision making, and in rehabilitation strategies. Although common statistic methods were applied (PCA and clustering), the new data scaling proposed allows avoiding errors of hiding joints with low ROM or noise magnification, providing subject-specific synergies, which are comparable since they do not depend on the ranges of motion recorded. In addition, Varimax rotation provides the sparsity in DoF, with a low computation cost.

## Supplementary information

Angles_24_healthy_subjects_24 ADLs.

## Data Availability

In methods section the steps followed have been thoroughly detailed in such a way that no specific custom-made code is needed to apply the method proposed and to reproduce the experiment.

## Data availability

The data that support the findings of this study are available as supplementary information files for open accessibility.
