# Publisher Correction: Sharing of hand kinematic synergies across subjects in daily living activities

**DOI:** 10.1038/s41598-020-68284-9

**Published:** 2020-07-01

**Authors:** Verónica Gracia-Ibáñez, Joaquín L. Sancho-Bru, Margarita Vergara, Néstor J. Jarque-Bou, Alba Roda-Sales

**Affiliations:** Department of Mechanical Engineering and Construction, Universitat Jaume I, Castelló, Spain

Correction to: *Scientific Reports* 10.1038/s41598-020-63092-7, published online 09 April 2020

The Article contains errors in Table 2 where some table headings were not marked as headings. The correct Table 2 appears below.

**Table 2:** Mean and SD values per joint to apply the non-standard scaling.

Digit	1				2–3	3–4	4–5	Palmar Arch
Joints	CMC A	CMC F	MCP F	IP F	MCP A			
Mean	7.8	8.0	2.6	18.8	7.3	4.9	5.4	41.1
SD	16.9	48.3	33.3	79.4	39.5	29.5	32.5	69.1

Digit	2	3	4	5	2	3	4	5
Joints	MCP F				PIP F			
Mean	22.7	27.0	25.3	23.3	52.5	45.0	46.5	41.1
SD	67.8	77.6	68.4	63.9	79.6	73.0	79.7	69.1

Digits 1 to 5 mean thumb to little digit. F for flexion, A for abduction.

